# Eye movements, not reaction times, reveal anticipatory attentional bias in childhood social anxiety disorder

**DOI:** 10.1111/jcpp.70115

**Published:** 2026-01-19

**Authors:** Nadine Vietmeier, Nik Dietze, Brunna Tuschen‐Caffier, Julia Asbrand

**Affiliations:** ^1^ Department of Psychology Humboldt‐Universität zu Berlin Berlin Germany; ^2^ University Clinic of Child and Adolescent Psychiatry and Psychotherapy, University Medical Centre EWL Bielefeld University Bielefeld Germany; ^3^ Department of Psychology Albert Ludwig University of Freiburg Freiburg im Breisgau Germany; ^4^ Department of Psychology Friedrich Schiller University Jena Jena Germany; ^5^ German Center for Mental Health (DZPG) Site Halle‐Jena‐Magdeburg Magdeburg Germany

**Keywords:** Hypervigilance, avoidance, attentional maintenance, children and adolescents, social phobia, gaze, eye‐tracking

## Abstract

**Background:**

Social anxiety disorder (SAD) is characterized by attentional biases that may contribute to its persistence. While adult models emphasize self‐focused and hypervigilant attention, there is limited understanding of how these processes operate in children. This study examined internal and external attentional biases in children with SAD during anticipation of a social stress task—a period when anxiety is typically elevated.

**Methods:**

Forty‐two children with a primary SAD diagnosis and 46 healthy controls (HC), aged 9–14 years, completed a reaction time (RT) task with internal (bodily) and external (visual) probes during anticipation of a speech task, while facing a peer video audience. RTs to probes and eye movements toward audience faces were recorded.

**Results:**

RTs did not differ between groups. Exploratory analyses revealed that age correlated negatively with RTs in both groups, suggesting developmental effects on processing speed, although no group differences in this relationship were found. Eye‐tracking revealed that children with SAD exhibited more frequent and longer fixations on audience faces during the initial phase of the task compared to HCs.

**Conclusions:**

Although RT tasks alone may not detect attentional biases in children with SAD, eye‐tracking indicated heightened attention to socially salient cues during anticipation. These findings highlight the importance of multimodal assessment to capture subtle hypervigilance in pediatric SAD.

## Introduction

Social anxiety disorder (SAD) is among the most common mental disorders in childhood and adolescence and significantly impairs social functioning and development (Burstein et al., 2011). It is characterized by an intense fear of negative evaluation and avoidance of social situations (Beidel & Turner, 2007). Prominent models of SAD (Clark & Wells, 1995; Rapee & Heimberg, 1997) identify attentional biases as key maintaining mechanisms alongside cognitive biases (e.g., post‐event processing; Asbrand et al., 2019). According to Clark and Wells (1995), heightened self‐focused attention increases awareness of internal states (e.g., thoughts, bodily sensations), distorting the processing of external social cues (e.g., observers' facial expressions). In contrast, Rapee and Heimberg (1997) propose hypervigilance toward both internal and external cues, further exacerbating anxiety. Together, these models describe two interrelated attentional foci: *internal attention*, that is, monitoring interoceptive or self‐referential signals, and *external attention*, that is, processing exteroceptive social cues (Deiters, Stevens, Hermann, & Gerlach, 2013; Mansell, Clark, & Ehlers, 2003). While well‐supported in adults, these mechanisms may differ in children due to developmental changes in cognition and emotion regulation (Halldorsson & Creswell, 2017).

Research on attentional biases in childhood SAD has primarily investigated internal and external biases separately. Internal biases have been linked to self‐focused attention (Bögels & Mansell, 2004), which has been shown to characterize children with SAD (Rapee et al., 2024). However, self‐focused attention is typically assessed via self‐report, mainly capturing cognitive aspects (e.g., thoughts and feelings about an upcoming situation; Harvey, Watkins, Mansell, & Shafran, 2004). In contrast, the assessment of perceptual aspects, such as the detection of subtle bodily cues (e.g., changes in heart rate; cf. Deiters et al., 2013), requires experimental paradigms such as reaction time (RT) tasks indexing responses to interoceptive stimuli (Nasiri et al., 2023). To the best of the authors' knowledge, RT paradigms have assessed self‐focused attention only in adults (e.g., Deiters et al., 2013; Mills, Grant, Judah, & White, 2014).

External biases are typically assessed using RT paradigms (e.g., dot‐probe tasks), and increasingly through eye‐tracking (Bögels & Mansell, 2004), yet findings remain mixed. RT studies suggest hypervigilance to threat in children with anxiety disorders in general, though effects vary with age and stimulus duration (Dudeney, Sharpe, & Hunt, 2015). Eye‐tracking studies show initial orienting to threat but often fail to differentiate anxious from non‐anxious groups (Lisk, Vaswani, Linetzky, Bar‐Haim, & Lau, 2020). Instead, children with anxiety disorders may avoid maintaining gaze on threat over time—especially in studies including mixed anxiety samples (Lisk et al., 2020). These discrepancies highlight the importance of multimodal assessment, particularly those combining RT and eye‐tracking measures (cf. Deiters et al., 2013).

Evidence specific to children with SAD remains inconsistent. RT studies report difficulty disengaging from threat (Pergamin‐Hight, Bitton, Pine, Fox, & Bar‐Haim, 2016) or hypervigilance depending on symptom severity (Waters, Mogg, Bradley, & Pine, 2011). Eye‐tracking work finds hypervigilance (Capriola‐Hall, Ollendick, & White, 2021; Schmidtendorf, Wiedau, Asbrand, Tuschen‐Caffier, & Heinrichs, 2018), effects limited to social‐stress contexts (Seefeldt, Krämer, Tuschen‐Caffier, & Heinrichs, 2014), age‐related differences (Hauffe, Rauschenbach, Fassot, Schmitz, & Tuschen‐Caffier, 2025), or influences of stimulus characteristics (Wieckowski, Capriola‐Hall, Elias, Ollendick, & White, 2019). Conversely, some studies indicate avoidance (Kleberg et al., 2017), vigilance‐avoidance patterns (Keil et al., 2018), delayed disengagement (Kleberg, Högström, Sundström, Frick, & Serlachius, 2021) or no bias (Högström et al., 2019). To explain these inconsistencies, Bögels and Mansell (2004) proposed already two decades ago that hypervigilance and subsequent avoidance, consistent with the vigilance‐avoidance model (Mogg & Bradley, 1998), are most evident under ecologically valid conditions.

Internal and external attention likely interact dynamically (Bögels & Mansell, 2004). Internal cues (e.g., bodily symptoms) may heighten vigilance and self‐focus (Clark & Wells, 1995). Physical symptoms can signal perceived “failure” in both adults (Gerlach, Mourlane, & Rist, 2004) and children with social anxiety (Schmitz, Blechert, Krämer, Asbrand, & Tuschen‐Caffier, 2012). Avoiding external threat may further amplify internal focus (Clark & Wells, 1995). Adult studies suggest that socially anxious individuals respond faster to internal but not external stimuli during stress anticipation (e.g., Deiters et al., 2013; Mills et al., 2014), suggesting anticipation as a critical period for attentional biases. Accordingly, Deiters et al. (2013) recommend combining RT paradigms with eye‐tracking to capture these dynamic, multifaceted processes.

To date, no study has investigated how internal and external biases manifest during anticipation of a social stressor in clinically diagnosed children. Investigating this interplay through multimodal approaches may reveal early, potentially modifiable mechanisms that maintain SAD in children, guiding both theory and intervention.

Collectively, theoretical and empirical evidence indicates that attentional biases in SAD involve not only external threat processing but also heightened monitoring of internal bodily states (Clark & Wells, 1995; Mansell et al., 2003). During anticipation of social evaluation, these biases may manifest as preferential attention to interoceptive cues and avoidance of external threat (Bögels & Mansell, 2004; Mogg & Bradley, 1998). Such anticipatory attentional patterns may therefore represent a key mechanism underlying anxiety maintenance in children.

### The current study

Despite increasing research on attentional biases in childhood SAD, several gaps remain. Most studies have examined internal or external biases in isolation and relied on single‐method assessments. Moreover, existing work largely concerns adults or subclinical samples, limiting relevance for clinically diagnosed children. The present study addresses these gaps by assessing both internal and external biases during anticipation of a social stress task, using RT and eye‐tracking measures.

We hypothesized that, during the anticipation phase, children with SAD would (1) exhibit faster RTs to internal stimuli, reflecting heightened attention to bodily sensations (Deiters et al., 2013; Mansell et al., 2003; Mills et al., 2014), and (2) demonstrate initial hypervigilance toward a potentially threatening stimulus (a neutral peer audience), followed by attentional avoidance, as indicated by gaze patterns (Bögels & Mansell, 2004; Lisk et al., 2020), relative to a healthy control (HC) group.

## Methods

### Ethical approvals and consent procedures

The study received ethical approval from the Ethics Committee of the University of Freiburg, Germany (application no. 24/19) and complied with the Declaration of Helsinki and institutional standards. Informed consent was obtained from all participants and their guardians following comprehensive written and verbal study explanations. Upon study completion, children received a €60 voucher, and guardians a €40 reimbursement. Children in the clinical group were offered treatment at the department's outpatient clinic or referred for additional services, as appropriate.

### Trial design

The study was registered with the German Clinical Trials Register (DRKS00018880). It formed part of a larger research project involving two laboratory sessions. The present analyses focused on the first session examining attentional biases during anticipation. Further procedural details are available elsewhere (Vietmeier, Tuschen‐Caffier, & Asbrand, 2025).^1^

As power analyses for mixed models are limited due to the complexity of their structures, our preregistered sample size was guided by a comparable study (*n* = 80; Schmidtendorf et al., 2018). Because our study formed part of a broader project with a required sample of *N* = 92, all eligible children were included to maximize statistical power.

### Participants

Children aged 9–14 years were recruited via population registers, schools, social media, and local newspapers. Inclusion criteria required a primary diagnosis of SAD (DSM‐5; American Psychiatric Association, 2013) for the SAD group and no current or lifetime diagnosis of any mental disorder for the HC group. Exclusion criteria were suspected or confirmed autism spectrum disorder, acute suicidal tendencies, intellectual disabilities (estimated IQ < 80, inferred from school placement), use of medications that could affect emotional or psychophysiological responses (e.g., anxiolytics), and sibling participation. Diagnostic assessment followed the supervision and consensus procedures described below.

Due to technical difficulties resulting in missing data for the RT tasks, three children were excluded from the original sample (cf. Vietmeier et al., 2025), resulting in a final sample of 42 children with SAD and 46 HC children. Participant characteristics are presented in Table 1. All participants were fluent in German and had normal (*n* = 69) or corrected‐to‐normal eye vision (*n* = 19). Comorbidities in the SAD group included: depressive disorders (*n* = 9), specific phobia (*n* = 7), separation anxiety disorder (*n* = 2), insomnia (*n* = 2), generalized anxiety disorder (*n* = 1), agoraphobia (*n* = 1), post‐traumatic stress disorder (*n* = 1), tic disorder (*n* = 1), enuresis (*n* = 1), and attention‐deficit/hyperactivity disorder (*n* = 1). No participant had received prior psychotherapy or medication for SAD, though one child was taking asthma medication.

**Table 1:** Participant characteristics

Characteristic	Group		Statistics
	SAD	HC	
Sample size (*n*)	42	46	
Mean age (*SD*), in years	12.33 (1.51)	11.89 (1.49)	ns^a^
Female (%)	69.0	50.0	ns^b^
Mean SDQ (*SD*)	15.79 (5.29)	8.53 (5.38)	*p* < .001^a^
Mean SDQ—mother (*SD*)	12.29 (5.30)	4.78 (4.05)	*p* < .001^a^
Mean SDQ—father (*SD*)	11.26 (5.62)	5.85 (4.61)	*p* < .001^a^
Mean SPAI‐C (*SD*)	30.25 (9.76)	7.95 (6.58)	*p* < .001^a^
Mean SASC‐R‐D (*SD*)	58.50 (13.55)	34.73 (10.05)	*p* < .001^a^
Mean SASC‐R‐D—mother (*SD*)	60.81 (11.10)	32.11 (10.06)	*p* < .001^a^
Mean SASC‐R‐D—father (*SD*)	52.74 (10.62)	35.49 (10.83)	*p* < .001^a^
Mean DIKJ (*SD*)	22.60 (10.33)	8.38 (5.66)	*p* < .001^a^
School^c^			
Elementary school (%)	28.6	37.0	ns^b^
Integrated secondary school (%)	11.9	13.0	ns^b^
Grammar school (%)	47.6	41.3	
Comprehensive school (%)	2.4	2.2	ns^b^
Other (%)	9.5	4.3	ns^b^
Not specified (%)	0.0	2.2	ns^b^
Child lives primarily with…^c^			
Both parents (%)	76.2	82.6	ns^b^
Mother (%)	14.3	10.9	ns^b^
Mother and female partner (%)	2.4	0.0	ns^b^
Mother and male partner (%)	7.1	2.2	ns^b^
Father and male partner (%)	0.0	2.2	ns^b^
Not specified (%)	0.0	2.2	ns^b^
Excluded from eye‐tracking^d^ (*n*)	10	9	ns^b^

SDQ = Strengths and Difficulties Questionnaire (Klasen et al., 2003), range: 0–40. SPAI‐C = Social Phobia and Anxiety Inventory for Children (Melfsen et al., 2011), range: 0–42. SASC‐R‐D = Social Anxiety Scale for Children‐Revised (Melfsen & Warnke, 2011), range: 18–90. DIKJ = Children's Depression Inventory (Stiensmeier‐Pelster et al., 2014), range: 0–56.

^a^ Based on *t* test.

^b^ Based on chi‐square test.

^c^ Based on maternal report.

^d^ Exclusion criteria described in Methods.

### Stimuli and measures

#### Diagnostic assessment

##### Diagnostic Interview for Mental Disorders in Children and Adolescents (Kinder‐DIPS; Schneider, Pflug, Margraf, & In‐Albon, 2017)

The Kinder‐DIPS is a structured interview based on DSM‐5 and International Classification of Diseases (ICD‐10; World Health Organization, 2004) criteria, conducted separately with child and parent. All interviews were conducted online due to the COVID‐19 pandemic, video‐recorded for quality assurance, and administered by a licensed child psychotherapist or a trained graduate psychology student under supervision. In cases of diagnostic uncertainty, a second licensed child and adolescent psychotherapist was consulted, and diagnoses were reached by consensus. The Kinder‐DIPS has demonstrated high interrater reliability (*κ* = .88–.98) and good test–retest reliability (75–100%; Schneider et al., 2017).

##### General psychopathology

General psychopathology was assessed through the German version (Klasen, Woerner, Rothenberger, & Goodman, 2003) of the Strengths and Difficulties Questionnaire (SDQ; Goodman, 1997), completed by children and parents. The 25‐item behavioral screening tool includes subscales for emotional symptoms, conduct problems, hyperactivity‐inattention, peer relationships, and prosocial behavior (five items on a 3‐point scale each). In this sample, internal consistencies for the subscales were moderate (child report: *α* = .583–.824, mother report: *α* = .564–.872, father report: *α* = .429–.831), consistent with the original publication (*α* = .55–.77; *r*_tt_ = .58–.67; Lohbeck, Schultheiß, Petermann, & Petermann, 2015).

##### Social anxiety symptoms

Social anxiety symptoms were measured using the 26‐item Social Phobia and Anxiety Inventory for Children (SPAI‐C, Beidel, Turner, Hamlin, & Morris, 2000; German version: Melfsen, Walitza, & Warnke, 2011), a self‐report tool with excellent internal consistency (current sample: *α* = .982), and the Social Anxiety Scale for Children‐Revised (SASC‐R‐D, La Greca & Stone, 1993; German version: Melfsen & Warnke, 2011), a 22‐item tool completed by children and parents, also with excellent internal consistency (current sample: child report: *α* = .951, mother report: *α* = .967, father report: *α* = .931). Both measures were included to increase construct validity and reduce single‐method bias (Silverman & Ollendick, 2005).

##### Depressive symptoms

Depressive symptoms were assessed using the Children's Depression Inventory (CDI; Kovacs, 2003; German version: Stiensmeier‐Pelster, Braune‐Krickau, Schürmann, & Duda, 2014), a validated 26‐item self‐report tool. In the current study, the internal consistency was excellent (*α* = .941).

#### Laboratory session assessment

##### Self‐reported anxiety

Children assessed their anxiety levels using a child‐friendly visual analog scale (VAS; Schmitz, Krämer, Blechert, & Tuschen‐Caffier, 2010; Schmitz, Krämer, & Tuschen‐Caffier, 2011). They marked a point on a 100 mm horizontal line anchored at “no anxiety” and “extreme anxiety”; responses were transformed from mm to a 0–10 scale for analysis.

##### RT task

**Figure 1:**
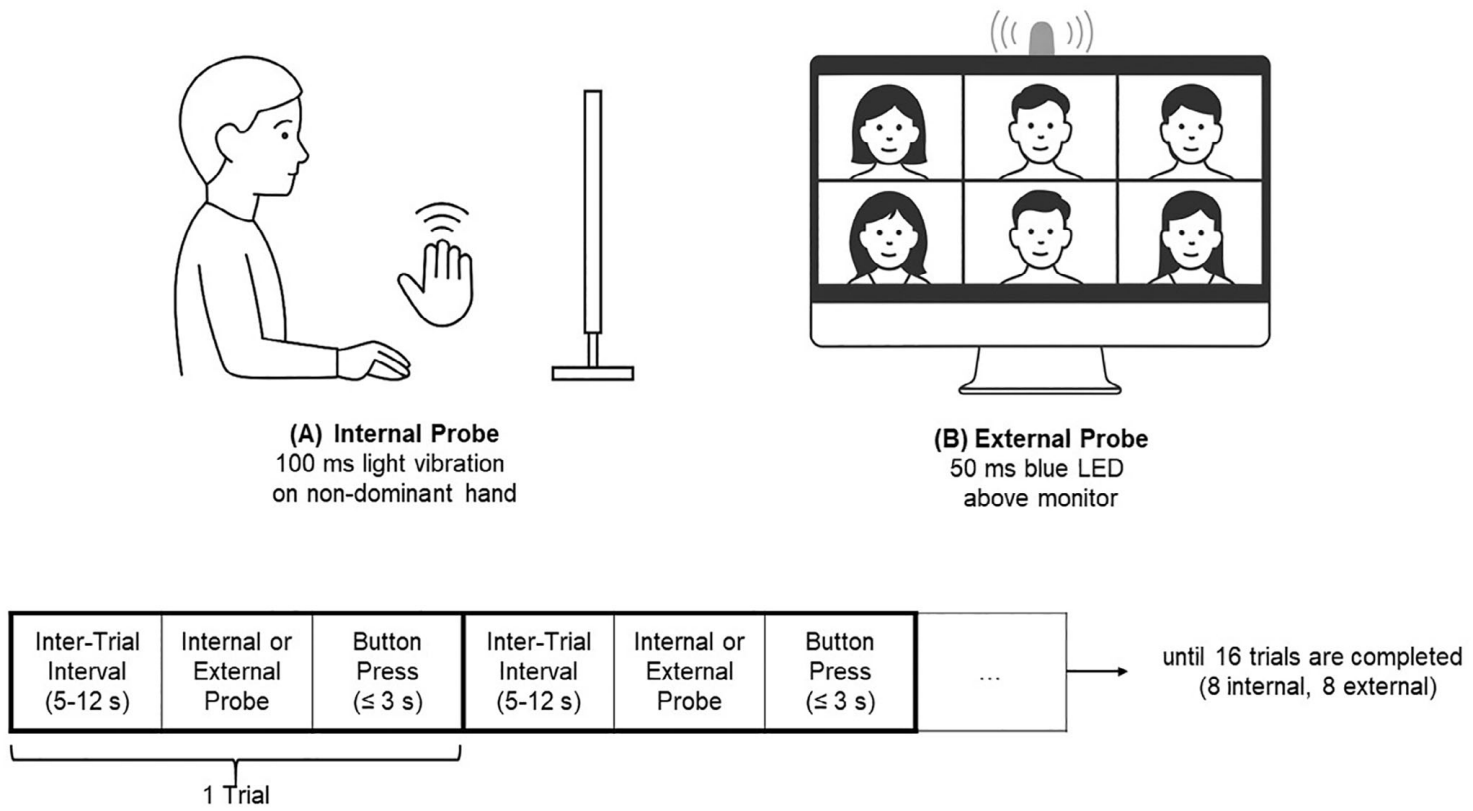
Schematic illustration of the RT task assessing internal and external attention. (A) A 100 ms vibration applied via the motor of a mobile phone on the non‐dominant hand represented an interoceptive bodily signal (internal probe). (B) A 50 ms blue LED (rendered in gray for print) above the monitor represented a neutral visual cue (external probe). Each boxed sequence represents one trial, which began with an inter‐trial interval of variable duration (5–12 s), followed by one probe (internal or external). Probe types were delivered in randomized and counterbalanced order (eight trials of each type). Participants had up to 3 s to respond, and reaction times (RTs), measured from probe onset to button press, indexed attentional focus

The RT task (adapted for use with children from Deiters et al., 2013; based on Mansell et al., 2003) contrasted responses to interoceptive (internal) and neutral exteroceptive (external) probes during anticipation of evaluation (Figure 1). The task was implemented in ePrime 3.0 (Psychology Software Tools, Inc., 2016). Participants pressed a button on the Chronos response device (Psychology Software Tools, Inc., 2015) when detecting either an internal probe (100 ms light vibration via a mobile phone motor on the non‐dominant hand) or an external probe (50 ms blue LED above the monitor).

Participants were informed that vibrations indicated changes in their pulse or hand sweating, thereby providing feedback on their internal physiological state. Specifically, they were told: “The device will sometimes vibrate briefly. It does so whenever the sensors on your chest or hand detect a noticeable bodily signal”. This explanation encouraged engagement with interoceptive cues (cf. Deiters et al., 2013; Mansell et al., 2003). Sensors were attached to enhance credibility. Children rated perceived vibration intensity on a scale ranging from 0 (*not at all*) to 10 (*very strong*).

Children then completed one short practice run consisting of two internal and two external trials; if questions or difficulties arose, the practice was repeated until the procedure was clearly understood. After the practice, the anticipation phase of the paradigm was conducted, during which the RT task took place. Although a subsequent speech phase followed as part of the broader experimental protocol, no RT measurement occurred during that phase.

Unlike Deiters et al. (2013), we presented a prerecorded neutral‐looking peer audience simulating a Zoom session (instead of a single spectator), ensuring ecological validity and COVID‐safe implementation. Consequently, whereas the LED in Deiters et al. (2013) was positioned on the spectator's forehead, we positioned the LED lamp centrally above the monitor displaying the audience. This setup enabled a clear distinction between the neutral stimulus (i.e., LED lamp) and the potentially threatening stimulus (i.e., neutral‐looking audience), thereby facilitating the simultaneous analysis of gaze patterns.

Each participant completed 16 trials (8 internal and 8 external) in randomized order, with inter‐trial intervals varying in whole‐second steps between 5 and 12 s. Each of the eight interval durations appeared once per probe type, randomly distributed within the sequence. Responses >3,000 ms were coded as errors. The task lasted approximately 3 min. The proportion of missings that were due to instances where participants failed to respond to a probe (SAD: 3.62%; HC: 4.92%) did not differ between groups, *t*(86) = 0.541, *p* = .590, *d* = 0.115, 95% CI [−1.020, 1.872]. Consistent with Deiters et al. (2013), RTs more than ±2 SDs from the individual mean were excluded (Ratcliff, 1993), removing 118 trials. One child with SAD and one HC child were excluded for failing to respond to any probes during the anticipation RT task, preventing mean RT computation.

##### Gaze patterns

Gaze was recorded using Tobii Pro Glasses 2 (50 Hz; Tobii AB, Danderyd, Sweden). Calibration was conducted immediately before and after the anticipation phase, allowing for the detection and correction of any calibration drift (Niehorster et al., 2020), with validation repeated if needed. Data were processed in Tobii Pro Lab (v1.181; Tobii AB, 2021), using the I‐VT Fixation filter (see Tobii Pro Lab manual; Tobii Pro AB, 2020). A trained rater blind to group status verified mappings. Nineteen participants were excluded due to insufficient gaze detection (SAD: *n* = 6; HC: *n* = 8), glasses (SAD: *n* = 2; HC: *n* = 1), insufficient recording time (SAD: *n* = 1; HC: *n* = 0), or refusal to wear the eye‐tracking glasses (SAD: *n* = 1; HC: *n* = 0). Exclusions were evenly distributed across groups, *χ* ^2^(1) = 0.23, *p* = .629, *φ* = −0.52. The final sample for eye‐tracking analyses comprised 32 children with SAD and 37 HCs.

Areas of interest (AoIs) were defined for all audience members' faces, aggregated into one composite AoI (“all faces”) and the LED lamp (“lamp”), which served as a control variable (see Figure S1). Primary gaze metrics were fixation count (number of fixations within each AoI) and dwell time (total gaze duration in ms, including saccades). Data were binned in 1‐s intervals over the 3‐min anticipation and analyzed independently of RT data.

### Experimental procedure

**Figure 2:**
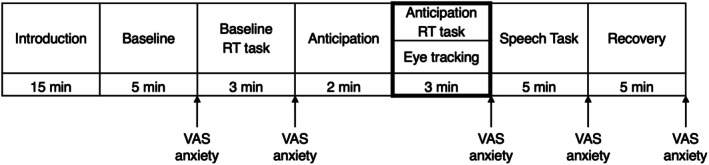
Procedure of laboratory session. For the present study and as preregistered, data from the anticipation phase with RT task were relevant (bold box). Eye‐tracking was assessed from baseline. Calibration and validation procedures took place before baseline, anticipation, and speech task. VAS anxiety = Retrospect state anxiety rating on a VAS scale (Schmitz et al., 2010, 2011)

Following diagnostic assessment, eligible children attended a 60–90‐min laboratory session (Figure 2). They were seated 60 cm from a 22‐inch computer screen in a room with constant lighting and controlled temperature. After a 5‐min baseline (slideshow of neutral landscape images; Asbrand et al., 2019), children completed the baseline RT task. During the subsequent anticipation phase, children prepared a 5‐min speech of their choice for a peer Zoom audience (cf. Miers, 2021; Westenberg et al., 2009). They were encouraged to make the speech “as interesting as possible” so that the “other children would want to get to know [them] better”. Participants had 2 min for initial preparation, followed by 3 min of additional preparation during which the anticipation RT task was completed. The prerecorded audience video was shown throughout to assess gaze behavior. The anticipation phase was followed by the speech itself and a recovery phase. Participants completed questionnaires at multiple timepoints (see Figure 2); only those relevant here are reported. Upon study completion, participants were thoroughly debriefed in an age‐appropriate manner and thanked for their participation. As part of the study design, two deceptions were used to enhance ecological validity: participants were told that the peer audience was watching them live, although the video was prerecorded, and that the light vibrations reflected changes in their pulse and hand sweating, although these signals were externally generated. Both procedures were approved by the ethics committee and fully explained during debriefing. No adverse reactions were reported by any participant.

### Statistical analyses

Statistical analyses were conducted in R (v4.1.2; R Core Team, 2021). A significance level of *α* = .05 was applied for all tests. For Hypothesis 1, between‐group effects were analyzed via repeated measures ANOVAs with group (SAD, HC) as a between‐factor, and time (baseline, anticipation) and stimulus type (internal, external) as within‐factors. Violations of sphericity were corrected using Greenhouse–Geisser. Significant interactions were decomposed via simple main effects. For Hypothesis 2, generalized (hit vs. no hit) and linear mixed‐effect models (RT in ms) were run using *lme4* (Bates, Mächler, Bolker, & Walker, 2015), with group and time bin (in 1‐s intervals) as fixed effects and participant as random intercept. To examine significant interactions, simple slope analyses were conducted using *emmeans* (Lenth & Piaskowski, 2025).

### Use of artificial intelligence tools

Parts of the manuscript text and Figure 1 were prepared with the assistance of OpenAI's ChatGPT (version GPT‐5) to improve language clarity and visualization. All AI‐generated content was critically reviewed, edited, and approved by the authors, who take full responsibility for the final version.

## Results

### Manipulation check

**Figure 3:**
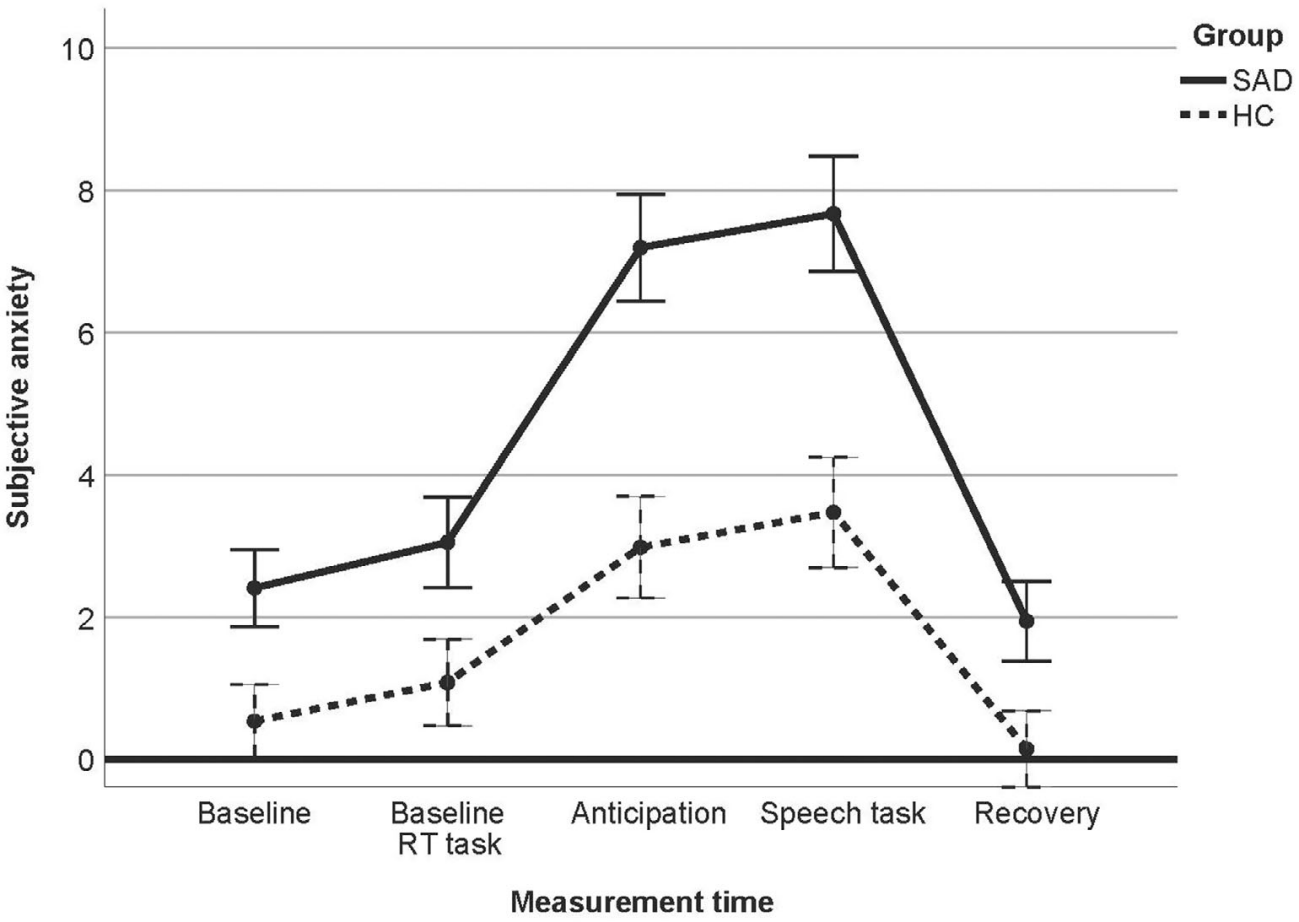
Group differences in self‐reported anxiety. HC, healthy control group; SAD, social anxiety disorder group. Error bars: 95% CI. Self‐reported anxiety was rated retrospectively on a VAS scale (range: 0–10; Schmitz et al., 2010, 2011). Children with SAD reported significantly more anxiety than HCs at all five measurement points (all *p*s < .001)

**Figure 4:**
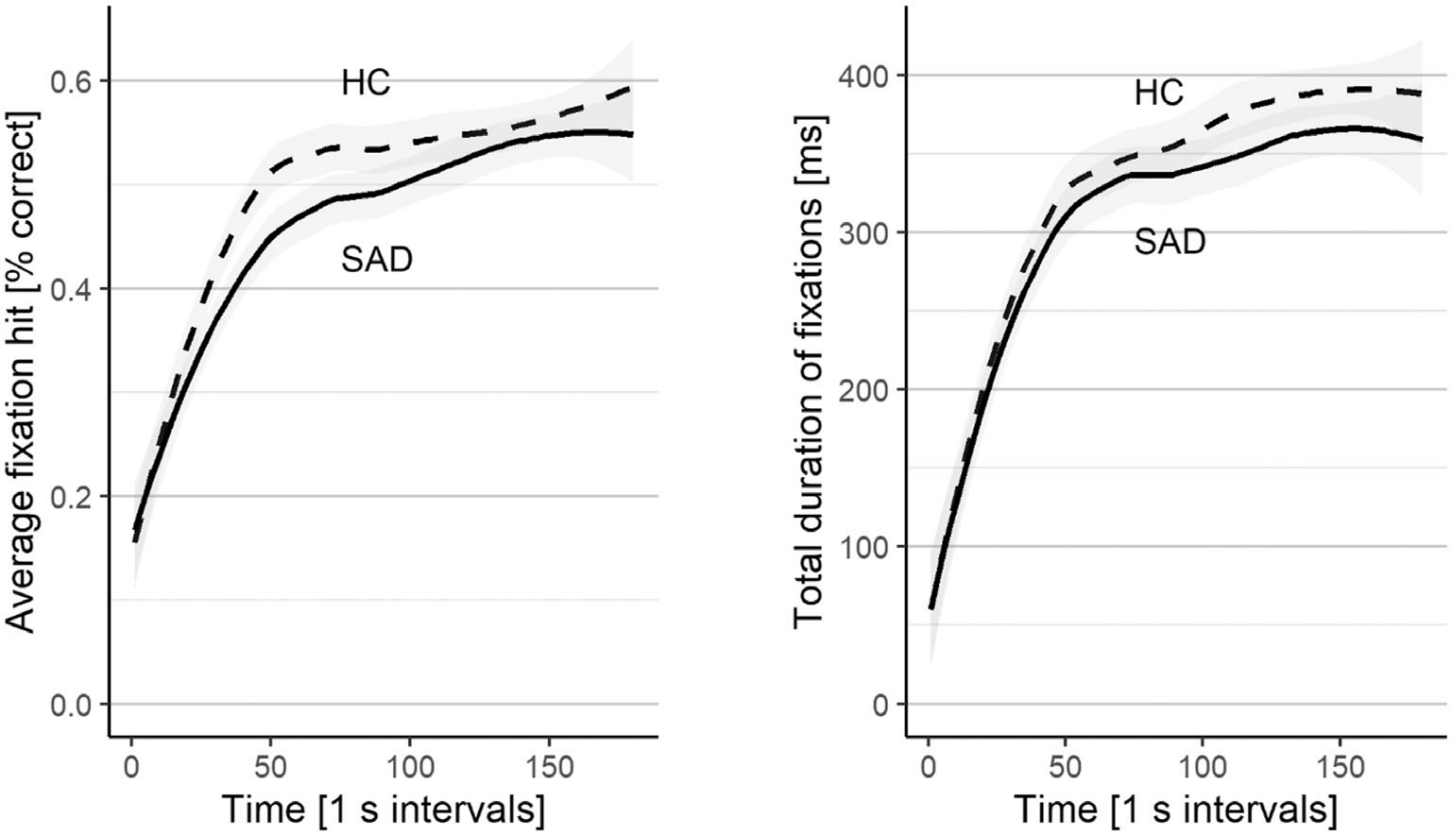
Gaze behavior patterns on lamp during anticipation with RT Task. HC, healthy control group; Lamp, LED lamp above the screen (external stimulus for the RT task); SAD, social anxiety disorder group. Children with SAD showed no difference in fixation numbers and durations

Children with SAD reported significantly higher anxiety than HCs across all time points, all *t*s ≥ −8.077, all *p*s < .001, all *d*s ≥ −1.709. In both groups, anxiety increased significantly from *baseline* to the *baseline RT task* and again from the *baseline RT task* to the *speech task* (SAD: all *t*s ≥ −12.307, all *p*s ≤ .012, all *d*s ≥ −1.899; HC: all *t*s ≥ −6.490, all *p*s < .007, all *d*s ≥ −0.957), confirming successful stress induction. Figure 3 illustrates anxiety trajectories over time. No group differences emerged in perceived internal probe intensity, *t*(78) = −0.111, *p* = .912, *d* = −0.025, 95% CI [−0.966, 0.864]. Gaze behavior toward the neutral external stimulus (LED) showed no significant effects of group or time (see Figure 4).

### Reaction times

A significant main effect of time emerged, *F*(1, 84) = 11.886, *p* < .001, $\eta_{p}^{2}$ = .124. No main effects for group or stimulus type, nor any interactions were found, all *p*s > .057. Post hoc comparisons indicated slower RTs during anticipation compared to baseline for internal stimuli, *t*(85) = −3.079, *p* = .003, *d* = −0.332, 95% CI [−0.548, −0.114], as well as external stimuli, *t*(85) = −3.666, *p* = .001, *d* = −0.395, 95% CI [−0.614, −0.175]. Descriptive RT statistics by group, phase, and stimulus type are presented in Table 2 (see also Figure S2).

**Table 2:** Reaction time means and standard deviations (in ms) by group, phase, and stimulus type

Phase	Stimulus type	SAD (*n* = 41)	HC (*n* = 45)	Total (*n* = 86)
		*M* (*SD*)	*M* (*SD*)	*M* (*SD*)
Baseline	Internal	389.61 (96.03)	458.30 (241.29)	425.56 (188.86)
	External	377.29 (93.79)	443.36 (239.61)	411.87 (186.98)
Anticipation	Internal	491.58 (291.01)	508.68 (273.44)	500.53 (280.41)
	External	474.85 (248.99)	496.44 (236.85)	486.15 (241.52)

Reaction times (RTs) reflect mean response latencies to internal (vibration, representing interoceptive bodily signal) and external (LED lamp, representing neutral visual cue) probes during baseline and anticipation phases. HC, healthy control group; SAD, social anxiety disorder group.

Given no RT group differences, exploratory analyses tested whether age (in months) accounted for response speed differences (e.g., Reinholdt‐Dunne, Mogg, Esbjorn, & Bradley, 2012). In both groups, age was significantly negatively correlated with RTs to internal (SAD: *r*(39) = −.410, *p* = .008; HC: *r*(43) = −.311, *p* = .038) and external probes (SAD: *r*(39) = −.502, *p* < .001; HC: *r*(43) = −.363, *p* = .014). To test whether these associations differed by group, we used linear mixed‐effects models with random intercepts for participants, including *z*‐standardized age, stimulus type, and group as fixed effects. Age was significantly associated with RTs, with older children responding faster overall (*β* = −77.50, *SE* = 27.52, *t*(87.25) = −2.82, *p* = .006). No significant main effects of group or stimulus type were found, and none of the interaction terms reached significance (all *p*s > .19). Thus, the relationship between age and RTs did not differ between groups or stimulus types.

### Gaze behavior

**Figure 5:**
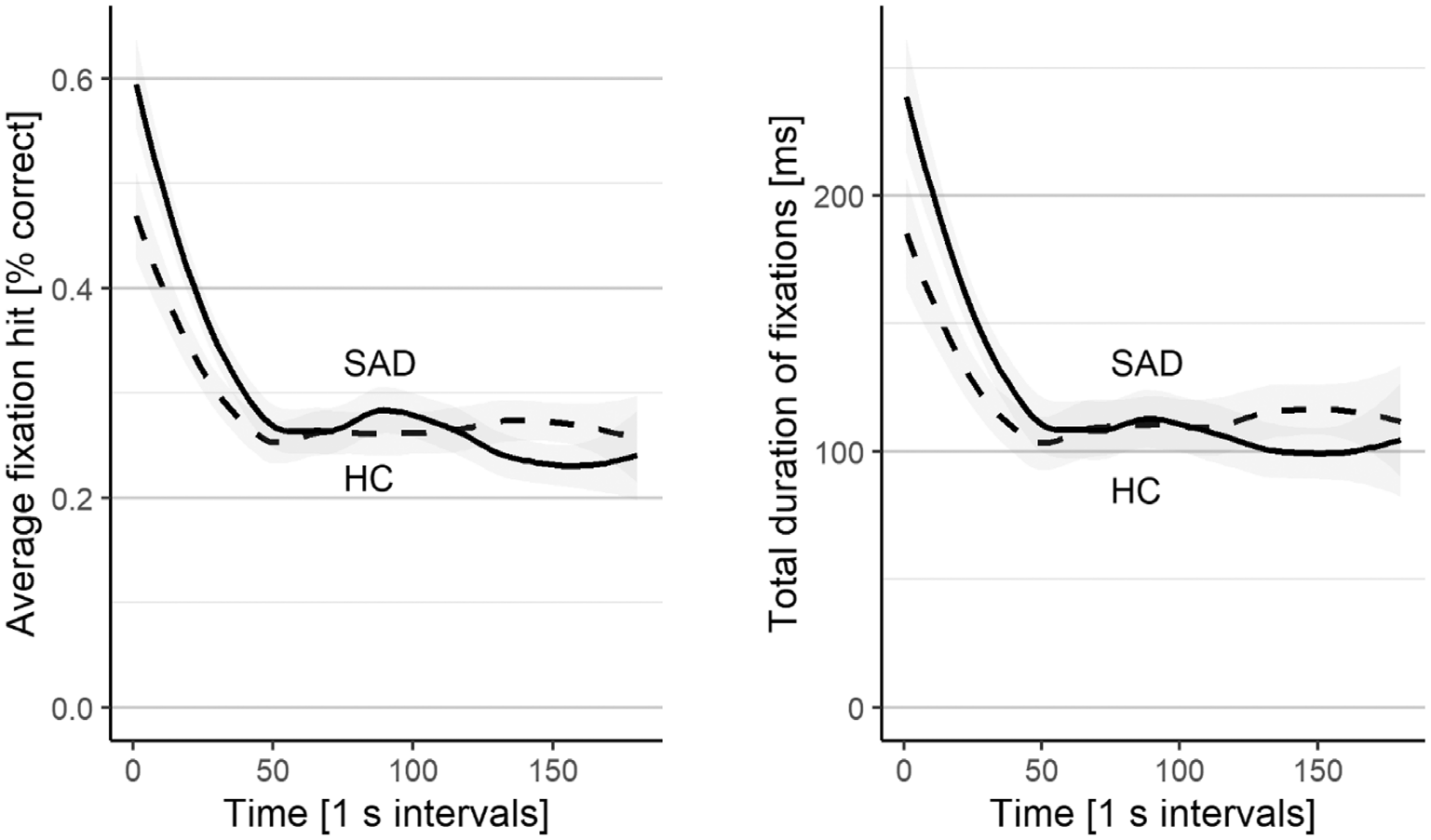
Gaze behavior patterns on audience during anticipation with RT task. HC, healthy control group; SAD, social anxiety disorder group. All faces = The facial regions of all children in the video audience (see Figure S1). During the initial phase of the task, children with SAD fixated on the audience more often and longer than HC children

Generalized and linear mixed‐effect models revealed significant main effects of time for both fixation hits, *β* = −0.16, *SE* = 0.03, *z* = −4.93, *p* < .001, and total fixation duration, *β* = −5.73, *SE* = 2.40, *t*(12349) = −2.39, *p* = .002, as well as significant group × time interactions for both fixation hits, *β* = −0.29, *SE* = 0.05, *z* = −5.83, *p* < .001, and total fixation duration on all faces, *β* = −17.25, *SE* = 3.46, *t*(12349) = −4.98, *p* < .001. Follow‐up simple slope analyses revealed that fixation probability decreased across time bins for both groups, but the decline was significantly steeper for children with SAD (*β* = −0.45, *z* = −12.48, *p* < .001, 95% CI [−0.52, −0.38]) than for HC children (*β* = −0.17, *z* = −4.93, *p* < .001, 95% CI [−0.23, −0.10]). A similar pattern emerged for total fixation duration, with children with SAD showing a steeper decline (*β* = −22.97, *z* = −9.18, *p* < .001, 95% CI [−27.90, −18.07]) than HC children (*β* = −5.73, *z* = 2.39, *p* = 0.02, 95% CI [−10.40, −1.03]). Figure 5 illustrates the trajectory in gaze behavior over time.

Exploratory analyses examined whether self‐reported social anxiety (SPAI‐C, SASC‐R‐D) predicted gaze indices. Neither measure was associated with fixation hits or total fixation duration (see Appendix S1 for full results).

## Discussion

This study examined internal and external attentional biases in children with SAD during anticipation of a social stress task, combining RT and eye‐tracking measures. As expected, the social‐evaluative context increased anticipatory anxiety, particularly in children with SAD. However, contrary to our hypothesis, no group differences emerged in RTs to internal or external stimuli. In contrast, eye‐tracking revealed enhanced early attention toward social stimuli in children with SAD, reflected in more frequent and longer fixations on the audience's faces during the initial phase of anticipation.

Several factors may explain the absence of RT group differences, despite previous adult findings (Deiters et al., 2013; Mansell et al., 2003; Mills et al., 2014). RT paradigms may miss subtle or dynamic attentional shifts, especially in children with still developing cognitive control (Halldorsson & Creswell, 2017). Age correlated negatively with RTs across groups, indicating that older children responded faster, possibly reflecting general improvements in processing speed rather than disorder‐specific effects (Dudeney et al., 2015; Field & Lester, 2010). Evidence accumulation models (Ratcliff, Smith, Brown, & McKoon, 2016) further suggest that RTs reflect not only attentional biases but also broader decision‐making processes. According to this framework, children with SAD may show heightened vigilance (faster accumulation due to threat sensitivity, i.e., drift rate), but also adopt more cautious decision‐making strategies (higher decision thresholds), potentially canceling RT differences. This interplay could obscure group differences in raw RTs, highlighting the need for computational modeling to uncover latent mechanisms (Parker & Ramsey, 2024). While such modeling was not feasible in our study due to task design limitations (Lerche, Voss, & Nagler, 2017; Parker & Ramsey, 2024), future studies could address this using binary‐choice paradigms with more trials. Alternatively, the visible audience may have directed attention outward, minimizing internal attention shifts and thus group differences in RTs.

Eye‐tracking results, by contrast, support models of external hypervigilance (Rapee & Heimberg, 1997), consistent with findings in socially anxious youth (Capriola‐Hall et al., 2021; Schmidtendorf et al., 2018), and meta‐analyses emphasizing child anxiety disorders (Bögels & Mansell, 2004; Lisk et al., 2020). The observed pattern fits the vigilance component of the vigilance‐avoidance model (Mogg & Bradley, 1998), indicating early orientation toward threat cues. The use of neutral (rather than overtly threatening) faces possibly prevented later avoidance, though neutral evaluators are known to evoke social‐evaluative threat (e.g., Trier Social Stress Test for Children, TSST‐C; Buske‐Kirschbaum et al., 1997). Future research might vary audience expressions (e.g., angry or disapproving; Dodd et al., 2015; Schmidtendorf et al., 2018) to test whether overtly negative cues amplify biases. Moreover, our focus on anticipation—a critical but understudied phase—may precede attentional avoidance onset (Kuckertz & Amir, 2015). Elevated anticipatory anxiety may impair disengagement from social cues (Schmidtendorf et al., 2018), underscoring the importance of temporally precise gaze analyses.

The discrepancy between RT and eye‐tracking measures suggests that they index distinct aspects of attentional processing. During anticipation, Rapee and Heimberg's (1997) model of external threat monitoring may better explain observable gaze behavior, while Clark and Wells's (1995) model of internal attention may capture processes not reflected in RTs. Eye‐tracking provides a more direct and reliable index of attentional bias than RT‐based tasks, which often show poor consistency (Clauss, Gorday, & Bardeen, 2022; Lazarov et al., 2019), particularly in youth samples (Lisk et al., 2020).

Clinically, although preliminary, our findings raise the possibility that interventions such as attention bias modification (Pergamin‐Hight, Pine, Fox, & Bar‐Haim, 2016) may be especially effective when targeting attention during anticipation, before maladaptive attentional loops are established.

Several limitations warrant consideration. First, although the overall sample size was comparable to prior work (Lidle & Schmitz, 2024; Schmidtendorf et al., 2018), eye‐tracking exclusions reduced statistical power and may limit generalizability. Building on our carefully diagnosed sample, future studies would benefit from larger samples. Second, the use of visual (external) versus tactile (internal) stimuli introduced a modality confound, limiting direct comparisons between internal and external attentional biases. Future research might display internal cues (e.g., heart rate on a monitor; Lin, Wen, Qian, He, & Zlomuzica, 2021) to permit direct comparison. Relatedly, vibrations were described as reflecting “noticeable bodily signals”, a child‐appropriate adaptation of Deiters et al.'s (2013) original instruction that vibrations signaled changes in pulse or sweating. This wording followed the established paradigm while avoiding overly suggestive explanations. Given that youth with SAD tend to overestimate and negatively evaluate bodily arousal (Asbrand, Schulz, Heinrichs, & Tuschen‐Caffier, 2020; Schmitz et al., 2012), and that anxiety is linked to increased attention to interoceptive cues (Clemente, Murphy, & Murphy, 2024), the task likely engaged processes central to SAD. Future research could systematically examine how different framings of interoceptive feedback influence attentional responding. Third, the anticipation phase remains under‐investigated, limiting comparability, while our findings underscore the value of time‐sensitive analyses. Fourth, the dual‐task demands of the paradigm (speech preparation plus RT task) likely increased cognitive load, possibly masking RT effects (cf. Deiters et al. 2013). However, such dual‐task demands may enhance ecological validity, as real‐world settings (e.g., classroom presentations) often require children to manage competing demands under stress. Additionally, the LED's placement above the audience display rather than in the interlocutor's face field, as in Deiters et al. (2013), may have reduced reciprocity cues (Lux et al., 2024). Moreover, participants were not presented with a self‐view, which might have attenuated the sense of being observed but at the same time raises the interesting question of whether adding such a feature could enhance self‐focused attention and perceived social evaluation. Importantly, the manipulation check confirmed robust increases in anticipatory anxiety, particularly in the SAD group, indicating that the task nevertheless elicited social threat successfully. Finally, the omission of physiological data prevented linking gaze patterns to arousal.

Our findings indicate early visual hypervigilance in children with SAD during anticipation of social evaluation, not captured by RT measures. This supports the value of ecologically valid, multimodal approaches to studying attentional biases in child anxiety. Integrating eye‐tracking, RT, and physiological measures—while considering developmental factors—may help identify attentional mechanisms underlying child SAD and inform targeted, developmentally sensitive interventions.

## Ethical considerations

The study received ethical approval from the Ethics Committee of the University of Freiburg, Germany, on 25 June 2019 (application no. 24/19) and complied with the Declaration of Helsinki and institutional standards. Informed consent was obtained from all participants and their guardians following comprehensive written and verbal study explanations.

**Key points**

**What's known?**

- Attentional biases are considered core maintaining mechanisms in childhood social anxiety disorder (SAD).
- Previous studies have primarily used reaction time (RT) paradigms, which may miss dynamic or early attentional shifts in children.

**What's new?**

- Eye‐tracking research has yielded inconsistent findings, potentially due to a lack of fine‐grained, time‐sensitive analyses or real‐world social contexts.
- Using a combined RT and eye‐tracking approach in an ecologically valid setting, this study found that children with SAD exhibit early hypervigilance toward peer faces during anticipation of social evaluation, not reflected in RT measures.

**What's relevant?**

- Results highlight the importance of multimodal, time‐sensitive assessment tools and suggest that early attentional processes may be critical targets for interventions in childhood SAD.

## Supporting information


**Figure S1.** Areas of interest (AoIs) for eye‐tracking analyses.
**Figure S2.** Mean RTs on external and internal stimuli during anticipation.
**Appendix S1.** Supplementary analysis.

## Data Availability

Data cannot be shared publicly as this is not included in the informed consent by participants, and the mental health data is particularly sensitive. However, deidentified participant data with annotations will be made available to other researchers upon reasonable request.

## Endnote

1 Additional results from this larger research project, including further findings from eye‐tracking data, assessments of children's social performance, and parental psychopathology, are currently being prepared for publication. Planned physiological measures (e.g., heart rate and skin conductance) were omitted due to technical issues.

## References

[jcpp70115-bib-0001] American Psychiatric Association . (2013). Diagnostic and statistical manual of mental disorders (5th edn). Author. Available from: 10.1176/appi.books.9780890425596 [last accessed 19 November 2024].

[jcpp70115-bib-0002] Asbrand, J. , Schmitz, J. , Krämer, M. , Nitschke, K. , Heinrichs, N. , & Tuschen‐Caffier, B. (2019). Effects of group‐based CBT on post‐event processing in children with social anxiety disorder following an experimental social stressor. Journal of Abnormal Child Psychology, 47, 1945–1956. 31073879 10.1007/s10802-019-00558-x

[jcpp70115-bib-0003] Asbrand, J. , Schulz, A. , Heinrichs, N. , & Tuschen‐Caffier, B. (2020). Biased perception of physiological arousal in child social anxiety disorder before and after cognitive behavioral treatment. Clinical Psychology in Europe, 2, e2691. 36397826 10.32872/cpe.v2i2.2691 PMC9645492

[jcpp70115-bib-0004] Bates, D. , Mächler, M. , Bolker, B. , & Walker, S. (2015). Fitting linear mixed‐effects models using lme4. Journal of Statistical Software, 67, 1–48.

[jcpp70115-bib-0005] Beidel, D.C. , & Turner, S.M. (2007). Shy children, phobic adults: Nature and treatment of social anxiety disorders (2nd edn pp. xiii, 398). Washington, DC: American Psychological Association.

[jcpp70115-bib-0006] Beidel, D.C. , Turner, S.M. , Hamlin, K. , & Morris, T.L. (2000). The Social Phobia and Anxiety Inventory for Children (SPAI‐C): External and discriminative validity. Behavior Therapy, 31, 75–87.

[jcpp70115-bib-0007] Bögels, S.M. , & Mansell, W. (2004). Attention processes in the maintenance and treatment of social phobia: Hypervigilance, avoidance and self‐focused attention. Clinical Psychology Review, 24, 827–856. 15501558 10.1016/j.cpr.2004.06.005

[jcpp70115-bib-0008] Burstein, M. , He, J.‐P. , Kattan, G. , Albano, A.M. , Avenevoli, S. , & Merikangas, K.R. (2011). Social phobia and subtypes in the National Comorbidity Survey–Adolescent Supplement: Prevalence, correlates, and comorbidity. Journal of the American Academy of Child and Adolescent Psychiatry, 50, 870–880. 21871369 10.1016/j.jaac.2011.06.005 PMC3164536

[jcpp70115-bib-0009] Buske‐Kirschbaum, A. , Jobst, S. , Wustmans, A. , Kirschbaum, C. , Rauh, W. , & Hellhammer, D. (1997). Attenuated free cortisol response to psychosocial stress in children with atopic dermatitis. Psychosomatic Medicine, 59, 419–426. 9251162 10.1097/00006842-199707000-00012

[jcpp70115-bib-0010] Capriola‐Hall, N.N. , Ollendick, T.H. , & White, S.W. (2021). Attention deployment to the eye region of emotional faces among adolescents with and without social anxiety disorder. Cognitive Therapy and Research, 45, 456–467. 34305207 10.1007/s10608-020-10169-2 PMC8297822

[jcpp70115-bib-0011] Clark, D.M. , & Wells, A. (1995). A cognitive model of social phobia. In Social phobia: Diagnosis, assessment, and treatment (pp. 69–93). New York: The Guilford Press.

[jcpp70115-bib-0012] Clauss, K. , Gorday, J.Y. , & Bardeen, J.R. (2022). Eye tracking evidence of threat‐related attentional bias in anxiety‐ and fear‐related disorders: A systematic review and meta‐analysis. Clinical Psychology Review, 93, 102142. 35279537 10.1016/j.cpr.2022.102142

[jcpp70115-bib-0013] Clemente, R. , Murphy, A. , & Murphy, J. (2024). The relationship between self‐reported interoception and anxiety: A systematic review and meta‐analysis. Neuroscience and Biobehavioral Reviews, 167, 105923. 39427810 10.1016/j.neubiorev.2024.105923

[jcpp70115-bib-0014] Deiters, D.D. , Stevens, S. , Hermann, C. , & Gerlach, A.L. (2013). Internal and external attention in speech anxiety. Journal of Behavior Therapy and Experimental Psychiatry, 44, 143–149. 23187114 10.1016/j.jbtep.2012.09.001

[jcpp70115-bib-0015] Dodd, H.F. , Hudson, J.L. , Williams, T. , Morris, T. , Lazarus, R.S. , & Byrow, Y. (2015). Anxiety and attentional bias in preschool‐aged children: An eyetracking study. Journal of Abnormal Child Psychology, 43, 1055–1065. 25434325 10.1007/s10802-014-9962-x

[jcpp70115-bib-0016] Dudeney, J. , Sharpe, L. , & Hunt, C. (2015). Attentional bias towards threatening stimuli in children with anxiety: A meta‐analysis. Clinical Psychology Review, 40, 66–75. 26071667 10.1016/j.cpr.2015.05.007

[jcpp70115-bib-0017] Field, A.P. , & Lester, K.J. (2010). Learning of information processing biases in anxious children and adolescents. In J.A. Hadwin & A.P. Field (Eds.), Information processing biases and anxiety (1st edn, pp. 253–278). Hoboken, NY: Wiley. https://onlinelibrary.wiley.com/doi/10.1002/9780470661468.ch11

[jcpp70115-bib-0018] Gerlach, A.L. , Mourlane, D. , & Rist, F. (2004). Public and private heart rate feedback in social phobia: A manipulation of anxiety visibility. Cognitive Behaviour Therapy, 33, 36–45. 15224627 10.1080/16506070310014682

[jcpp70115-bib-0019] Goodman, R. (1997). The strengths and difficulties questionnaire: A research note. Journal of Child Psychology and Psychiatry, 38, 581–586. 9255702 10.1111/j.1469-7610.1997.tb01545.x

[jcpp70115-bib-0020] Halldorsson, B. , & Creswell, C. (2017). Social anxiety in pre‐adolescent children: What do we know about maintenance? Behaviour Research and Therapy, 99, 19–36. 28881221 10.1016/j.brat.2017.08.013

[jcpp70115-bib-0021] Harvey, A. , Watkins, E. , Mansell, W. , & Shafran, R. (2004). Cognitive Behavioural processes across psychological disorders: A transdiagnostic approach to research and treatment. Oxford: Oxford University Press.

[jcpp70115-bib-0022] Hauffe, V. , Rauschenbach, A.‐L. , Fassot, E.‐M. , Schmitz, J. , & Tuschen‐Caffier, B. (2025). Early hypervigilance and sustained attention for the eye region in adolescents with social anxiety disorder. Journal of Anxiety Disorders, 112, 103016. 40220702 10.1016/j.janxdis.2025.103016

[jcpp70115-bib-0023] Högström, J. , Nordh, M. , Larson Lindal, M. , Taylor, E. , Serlachius, E. , & Lundin Kleberg, J. (2019). Visual attention to emotional faces in adolescents with social anxiety disorder. PLoS One, 14, e0225603. 31756240 10.1371/journal.pone.0225603 PMC6874383

[jcpp70115-bib-0024] Keil, V. , Hepach, R. , Vierrath, S. , Caffier, D. , Tuschen‐Caffier, B. , Klein, C. , & Schmitz, J. (2018). Children with social anxiety disorder show blunted pupillary reactivity and altered eye contact processing in response to emotional faces: Insights from pupillometry and eye movements. Journal of Anxiety Disorders, 58, 61–69. 30053635 10.1016/j.janxdis.2018.07.001

[jcpp70115-bib-0025] Klasen, H. , Woerner, W. , Rothenberger, A. , & Goodman, R. (2003). Die deutsche Fassung des Strengths and Difficulties Questionnaire (SDQ‐Deu)—Übersicht und Bewertung erster Validierungs‐ und Normierungsbefunde. Available from: https://www.psycharchives.org/en/item/86f7e970‐a515‐4eb6‐84e2‐e942bda4d051 [last accessed 19 November 2024]. 14526759

[jcpp70115-bib-0026] Kleberg, J.L. , Högström, J. , Nord, M. , Bölte, S. , Serlachius, E. , & Falck‐Ytter, T. (2017). Autistic traits and symptoms of social anxiety are differentially related to attention to others' eyes in social anxiety disorder. Journal of Autism and Developmental Disorders, 47, 3814–3821. 28000078 10.1007/s10803-016-2978-z PMC5676829

[jcpp70115-bib-0027] Kleberg, J.L. , Högström, J. , Sundström, K. , Frick, A. , & Serlachius, E. (2021). Delayed gaze shifts away from others' eyes in children and adolescents with social anxiety disorder. Journal of Affective Disorders, 278, 280–287. 32977266 10.1016/j.jad.2020.09.022

[jcpp70115-bib-0028] Kovacs, M. (2003). The Children's Depression Inventory. Technical manual update (1st edn). Toronto, Canada: Multi‐Health Systems.

[jcpp70115-bib-0029] Kuckertz, J.M. , & Amir, N. (2015). Attention bias modification for anxiety and phobias: Current status and future directions. Current Psychiatry Reports, 17, 9. 25620791 10.1007/s11920-014-0545-x

[jcpp70115-bib-0030] La Greca, A.M. , & Stone, W.L. (1993). Social anxiety scale for children‐revised: Factor structure and concurrent validity. Journal of Clinical Child Psychology, 22, 17–27.

[jcpp70115-bib-0031] Lazarov, A. , Suarez‐Jimenez, B. , Tamman, A. , Falzon, L. , Zhu, X. , Edmondson, D.E. , & Neria, Y. (2019). Attention to threat in posttraumatic stress disorder as indexed by eye‐tracking indices: A systematic review. Psychological Medicine, 49, 705–726. 30178728 10.1017/S0033291718002313 PMC6399079

[jcpp70115-bib-0032] Lenth, R.V. , & Piaskowski, J. (2025). emmeans: Estimated Marginal Means, aka Least‐Squares Means. R package. Available from: https://rvlenth.github.io/emmeans/

[jcpp70115-bib-0033] Lerche, V. , Voss, A. , & Nagler, M. (2017). How many trials are required for parameter estimation in diffusion modeling? A comparison of different optimization criteria. Behavior Research Methods, 49, 513–537. 27287445 10.3758/s13428-016-0740-2

[jcpp70115-bib-0034] Lidle, L.R. , & Schmitz, J. (2024). Assessing visual avoidance of faces during real‐life social stress in children with social anxiety disorder: A mobile eye‐tracking study. Child Psychiatry & Human Development, 55, 24–35. 35708796 10.1007/s10578-022-01383-y PMC10796484

[jcpp70115-bib-0035] Lin, M. , Wen, X. , Qian, M. , He, D. , & Zlomuzica, A. (2021). Self‐focused attention vs. negative attentional bias during public speech task in socially anxious individuals. Behaviour Research and Therapy, 136, 103766. 33253981 10.1016/j.brat.2020.103766

[jcpp70115-bib-0036] Lisk, S. , Vaswani, A. , Linetzky, M. , Bar‐Haim, Y. , & Lau, J.Y.F. (2020). Systematic review and meta‐analysis: Eye‐tracking of attention to threat in child and adolescent anxiety. Journal of the American Academy of Child & Adolescent Psychiatry, 59, 88–99.e1. 31265874 10.1016/j.jaac.2019.06.006

[jcpp70115-bib-0037] Lohbeck, A. , Schultheiß, J. , Petermann, F. , & Petermann, U. (2015). Die deutsche Selbstbeurteilungsversion des strengths and difficulties questionnaire (SDQ‐Deu‐S): Psychometrische eigenschaften, faktorenstruktur und grenzwerte. [The German self‐report version of the strengths and difficulties questionnaire (SDQ‐Deu‐S): Psychometric properties, factor structure, and critical values.]. Diagnostica, 61, 222–235.

[jcpp70115-bib-0038] Lux, M. , Rabung, S. , Herrmann, P. , Albert, T. , Andreas, S. , & Hohnwald, S. (2024). Looking beyond the screen: Natural eye contact as a key to relatedness in teleconferences. Proceedings of the ACM Multimedia Systems Conference 2024 on ZZZ (pp. 499–502). Presented at the MMSys ‘24: ACM Multimedia Systems Conference 2024, Bari Italy: ACM. Available from: https://dl.acm.org/doi/10.1145/3625468.3652199 [last accessed 2 October 2025].

[jcpp70115-bib-0039] Mansell, W. , Clark, D.M. , & Ehlers, A. (2003). Internal versus external attention in social anxiety: An investigation using a novel paradigm. Behaviour Research and Therapy, 41, 555–572. 12711264 10.1016/s0005-7967(02)00029-3

[jcpp70115-bib-0041] Melfsen, S. , & Warnke, A. (2011). SASC‐R‐D – Social anxiety scale for children revised – Deutsche version. In C. Barkmann , M. Schulte‐Markwort , & E. Brähler (Eds.), Klinisch‐psychiatrische Ratingskalen für das Kindes‐ und Jugendalter, Diagnostik für Klinik und Praxis (Vol. 6, pp. 406–410). Göttingen, Germany: Hogrefe.

[jcpp70115-bib-0040] Melfsen, S. , Walitza, S. , & Warnke, A. (2011). Psychometrische Eigenschaften und Normierung des Sozialphobie und ‐angstinventars für Kinder (SPAIK) an einer klinischen Stichprobe. Zeitschrift für Kinder‐ und Jugendpsychiatrie und Psychotherapie, 39, 399–407. 22031012 10.1024/1422-4917/a000138

[jcpp70115-bib-0042] Miers, A.C. (2021). An investigation into the influence of positive peer feedback on self‐relevant cognitions in social anxiety. Behaviour Change, 38, 193–207.

[jcpp70115-bib-0043] Mills, A.C. , Grant, D.M. , Judah, M.R. , & White, E.J. (2014). The influence of anticipatory processing on attentional biases in social anxiety. Behavior Therapy, 45, 720–729. 25022782 10.1016/j.beth.2014.04.004

[jcpp70115-bib-0044] Mogg, K. , & Bradley, B.P. (1998). A cognitive‐motivational analysis of anxiety. Behaviour Research and Therapy, 36, 809–848. 9701859 10.1016/s0005-7967(98)00063-1

[jcpp70115-bib-0045] Nasiri, E. , Khalilzad, M. , Hakimzadeh, Z. , Isari, A. , Faryabi‐Yousefabad, S. , Sadigh‐Eteghad, S. , & Naseri, A. (2023). A comprehensive review of attention tests: Can we assess what we exactly do not understand? The Egyptian Journal of Neurology, Psychiatry and Neurosurgery, 59, 26.

[jcpp70115-bib-0046] Niehorster, D.C. , Santini, T. , Hessels, R.S. , Hooge, I.T.C. , Kasneci, E. , & Nyström, M. (2020). The impact of slippage on the data quality of head‐worn eye trackers. Behavior Research Methods, 52, 1140–1160. 31898290 10.3758/s13428-019-01307-0 PMC7280360

[jcpp70115-bib-0047] Parker, S. , & Ramsey, R. (2024). What can evidence accumulation modelling tell us about human social cognition? Quarterly Journal of Experimental Psychology, 77, 639–655. 10.1177/17470218231176950 PMC10880422 37154622

[jcpp70115-bib-0048] Pergamin‐Hight, L. , Bitton, S. , Pine, D.S. , Fox, N.A. , & Bar‐Haim, Y. (2016). Attention and interpretation biases and attention control in youth with social anxiety disorder. Journal of Experimental Psychopathology, 7, 484–498.

[jcpp70115-bib-0049] Pergamin‐Hight, L. , Pine, D.S. , Fox, N.A. , & Bar‐Haim, Y. (2016). Attention bias modification for youth with social anxiety disorder. Journal of Child Psychology and Psychiatry, 57, 1317–1325. 27435286 10.1111/jcpp.12599

[jcpp70115-bib-0050] Psychology Software Tools, Inc . (2015). Chronos. Sharpsburg, PA: Psychology Software Tools, Inc.

[jcpp70115-bib-0051] Psychology Software Tools, Inc . (2016). E‐Prime 3.0. https://support.pstnet.com

[jcpp70115-bib-0052] R Core Team . (2021). R statistical software. Vienna, Austria: R Foundation for Statistical Computing. https://www.R‐project.org

[jcpp70115-bib-0053] Rapee, R.M. , & Heimberg, R.G. (1997). A cognitive‐behavioral model of anxiety in social phobia. Behaviour Research and Therapy, 35, 741–756. 9256517 10.1016/s0005-7967(97)00022-3

[jcpp70115-bib-0054] Rapee, R.M. , McLellan, L.F. , Carl, T. , Hudson, J.L. , Parker, E. , Trompeter, N. , & Wuthrich, V.M. (2024). Testing theoretical processes that maintain paediatric social anxiety: A comparison between children and adolescents with social anxiety disorder, other mental disorders, and non‐clinical controls. Behaviour Research and Therapy, 183, 104638. 39321473 10.1016/j.brat.2024.104638

[jcpp70115-bib-0055] Ratcliff, R. (1993). Methods for dealing with reaction time outliers. Psychological Bulletin, 114, 510–532. 8272468 10.1037/0033-2909.114.3.510

[jcpp70115-bib-0056] Ratcliff, R. , Smith, P.L. , Brown, S.D. , & McKoon, G. (2016). Diffusion decision model: Current issues and history. Trends in Cognitive Sciences, 20, 260–281. 26952739 10.1016/j.tics.2016.01.007 PMC4928591

[jcpp70115-bib-0057] Reinholdt‐Dunne, M.L. , Mogg, K. , Esbjorn, B.H. , & Bradley, B.P. (2012). Effects of age and anxiety on processing threat cues in healthy children. Journal of Experimental Psychopathology, 3, 30–41.

[jcpp70115-bib-0058] Schmidtendorf, S. , Wiedau, S. , Asbrand, J. , Tuschen‐Caffier, B. , & Heinrichs, N. (2018). Attentional bias in children with social anxiety disorder. Cognitive Therapy and Research, 42, 273–288.

[jcpp70115-bib-0059] Schmitz, J. , Blechert, J. , Krämer, M. , Asbrand, J. , & Tuschen‐Caffier, B. (2012). Biased perception and interpretation of bodily anxiety symptoms in childhood social anxiety. Journal of Clinical Child & Adolescent Psychology, 41, 92–102. 22233249 10.1080/15374416.2012.632349

[jcpp70115-bib-0061] Schmitz, J. , Krämer, M. , & Tuschen‐Caffier, B. (2011). Negative post‐event processing and decreased self‐appraisals of performance following social stress in childhood social anxiety: An experimental study. Behaviour Research and Therapy, 49, 789–795. 21930262 10.1016/j.brat.2011.09.001

[jcpp70115-bib-0060] Schmitz, J. , Krämer, M. , Blechert, J. , & Tuschen‐Caffier, B. (2010). Post‐event processing in children with social phobia. Journal of Abnormal Child Psychology, 38, 911–919. 20496109 10.1007/s10802-010-9421-2

[jcpp70115-bib-0062] Schneider, S. , Pflug, V. , Margraf, J. , & In‐Albon, T. (2017). Kinder‐DIPS: Diagnostisches Interview bei psychischen Störungen im Kindes‐ und Jugendalter. Ruhr‐Universität Bochum (RUB). Available from: https://omp.ub.rub.de/index.php/RUB/catalog/book/101 [last accessed 19 November 2024].

[jcpp70115-bib-0072] Seefeldt, W. L. , Krämer, M. , Tuschen‐Caffier, B. , & Heinrichs, N. (2014). Hypervigilance and avoidance in visual attention in children with social phobia. Journal of behavior therapy and experimental psychiatry, 45(1), 105–112. 10.1016/j.jbtep.2013.09.004 24103693

[jcpp70115-bib-0063] Silverman, W.K. , & Ollendick, T.H. (2005). Evidence‐based assessment of anxiety and its disorders in children and adolescents. Journal of Clinical Child & Adolescent Psychology, 34, 380–411. 16026211 10.1207/s15374424jccp3403_2

[jcpp70115-bib-0064] Stiensmeier‐Pelster, J. , Braune‐Krickau, M. , Schürmann, M. , & Duda, K. (2014). Depressionsinventar für Kinder und Jugendliche (3rd edn). Göttingen: Hogrefe.

[jcpp70115-bib-0065] Tobii AB . (2021). Tobii Pro Lab analyzer edition. Danderyd, Sweden: Tobii AB.

[jcpp70115-bib-0066] Tobii Pro AB . (2020). Tobii Pro Lab user manual (version 1.152.1). Danderyd, Sweden: Tobii Pro AB.

[jcpp70115-bib-0067] Vietmeier, N. , Tuschen‐Caffier, B. , & Asbrand, J. (2025). Social stress task with parental support or self‐instruction decreases negative cognitions in children with social anxiety disorder. Scientific Reports, 15, 10220. 40133558 10.1038/s41598-025-95032-8 PMC11937552

[jcpp70115-bib-0068] Waters, A.M. , Mogg, K. , Bradley, B.P. , & Pine, D.S. (2011). Attention bias for angry faces in children with social phobia. Journal of Experimental Psychopathology, 2, 475–489.

[jcpp70115-bib-0069] Westenberg, P.M. , Bokhorst, C.L. , Miers, A.C. , Sumter, S.R. , Kallen, V.L. , van Pelt, J. , & Blöte, A.W. (2009). A prepared speech in front of a pre‐recorded audience: Subjective, physiological, and neuroendocrine responses to the Leiden public speaking task. Biological Psychology, 82, 116–124. 19576261 10.1016/j.biopsycho.2009.06.005

[jcpp70115-bib-0070] Wieckowski, A.T. , Capriola‐Hall, N.N. , Elias, R. , Ollendick, T.H. , & White, S.W. (2019). Variability of attention bias in socially anxious adolescents: Differences in fixation duration toward adult and adolescent face stimuli. Cognition and Emotion, 33, 825–831. 29774787 10.1080/02699931.2018.1476322 PMC6358515

[jcpp70115-bib-0071] World Health Organization (Ed.). (2004). International statistical classification of diseases and related health problems (10th revision, 2nd edn). Geneva: World Health Organization. 10.2471/BLT.25.294650 PMC13531092 42683092

