# Supplementary material for: Eye movements, not reaction times, reveal anticipatory attentional bias in childhood social anxiety disorder
Source: J Child Psychol Psychiatry. 2026 Jan 19;67(7):1073–84. doi: 10.1111/jcpp.70115 (PMC13265622; doi:10.1111/jcpp.70115)
Supplement: Supplementary file 1 — Figure S1. Areas of interest (AoIs) for eye‐tracking analyses. Figure S2. Mean RTs on external and internal stimuli during anticipation. Appendix S1. Supplementary analysis. [file JCPP-67-1073-s001.docx]

**Eye Movements, Not Reaction Times, Reveal Anticipatory Attentional Bias in Childhood Social Anxiety Disorder**

**Supporting Information**

**Figure S1**

*Areas of Interest (AoIs) for Eye-tracking Analyses*

*
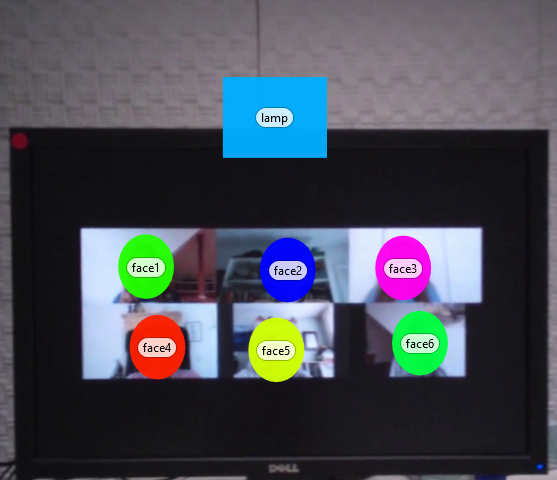
*

*Note.* All audience member’s faces (“face1” to “face6”) were aggregated into a single AoI (“all faces”). The LED lamp AoI (“lamp”; external stimulus for the RT task) was used as a manipulation check.

**Figure S2**

*Mean RTs on External and Internal Stimuli During Anticipation*


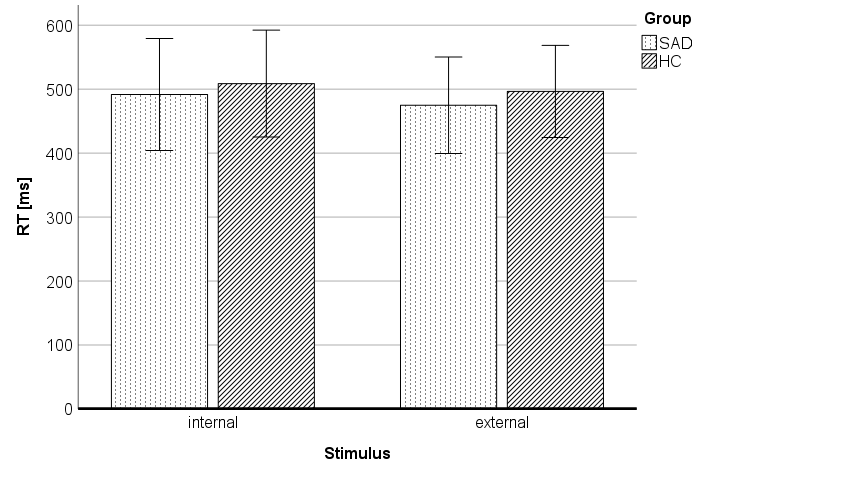


*Note.* SAD = Social anxiety disorder group. HC = Healthy control group. RTs = Reaction Times. Error bars: 95% CI. Groups did neither differ in their RTs on internal nor external stimuli during anticipation.

**Appendix S1. Supplementary Analysis**

To examine whether individual differences in self-reported social anxiety were associated with gaze behavior, exploratory models were run including total scores from both social anxiety questionnaires (SPAI-C and SASC-R-D) as additional predictors. These models extended the generalized and linear mixed-effects models described in the main results section for fixation hits and total fixation duration.

Across models, time significantly predicted fixation hits, β = –0.12, SE = 0.03, z = –3.56, p < .001. The interaction between group and time was also significant for fixation hits (β = –0.33, SE = 0.05, z = –6.64, p < .001) and for total fixation duration (β = –19.49, SE = 3.48, t(12170) = –5.61, p < .001).

In contrast, neither the SPAI-C nor the SASC-R-D total scores were significant predictors of gaze behavior. Specifically, SPAI-C scores did not predict fixation hits (β = 0.02, SE = 0.04, z = 0.61, p = .54) or total fixation duration (β = 2.32, SE = 3.17, t(64) = 0.74, p = .47). Likewise, SASC-R-D scores were not significantly associated with fixation hits (β = –0.03, SE = 0.03, z = –1.21, p = .22) or total fixation duration (β = –2.36, SE = 2.31, t(64) = –1.02, p = .31).

Taken together, these results indicate that while temporal dynamics and group differences significantly influenced gaze behavior, self-reported symptom severity did not account for additional variance in fixation frequency or duration.
